# High-throughput ChIPmentation: freely scalable, single day ChIPseq data generation from very low cell-numbers

**DOI:** 10.1186/s12864-018-5299-0

**Published:** 2019-01-18

**Authors:** Charlotte Gustafsson, Ayla De Paepe, Christian Schmidl, Robert Månsson

**Affiliations:** 1 Center for Hematology and Regenerative Medicine Huddinge, Karolinska Institutet, Stockholm, Sweden; 2 Hematology Center, Karolinska University Hospital, Stockholm, Sweden; 3 Regensburg Centre for Interventional Immunology (RCI) and University Medical Center, Regensburg, Germany

**Keywords:** Chromatin immunoprecipitation, ChIP-seq, ChIPmentation, High-throughput genomics, Epigenetics

## Abstract

**Background:**

Chromatin immunoprecipitation coupled to sequencing (ChIP-seq) is widely used to map histone modifications and transcription factor binding on a genome-wide level.

**Results:**

We present high-throughput ChIPmentation (HT-ChIPmentation) that eliminates the need for DNA purification prior to library amplification and reduces reverse-crosslinking time from hours to minutes.

**Conclusions:**

The resulting workflow is easily established, extremely rapid, and compatible with requirements for very low numbers of FACS sorted cells, high-throughput applications and single day data generation.

**Electronic supplementary material:**

The online version of this article (10.1186/s12864-018-5299-0) contains supplementary material, which is available to authorized users.

## Background

The combination of chromatin immunoprecipitation with high-throughput sequencing (ChIP-seq) has become the method of choice for mapping chromatin-associated proteins and histone-modifications on a genome-wide level.

The ChIP-seq methodology has rapidly developed [1–4]. Despite this, performing ChIP-seq on limited cell-numbers and in a high-throughput manner remains technically challenging. This is largely due to decreasing input material leading to progressively increasing losses of material during DNA preparation and inefficiencies of enzymatic reactions used for library preparation. While elegant strategies have been developed to resolve these issues, they remain laborious and have not seen wider use [5–12].

ChIPmentation [3] effectively alleviates the issues associated with traditional library preparation methodologies by introducing sequencing-compatible adapters to bead-bound chromatin using Tn5 transposase (tagmentation). While fast and convenient, the methodology still relies on the use of traditional reverse crosslinking and DNA purification procedures prior to library amplification, hampering processing time, DNA recovery, and limiting scalability for high-throughput applications.

Here, we present freely scalable high-throughput ChIPmentation (HT-ChIPmentation) that by eliminating the need for DNA purification and traditional reverse-crosslinking prior to library amplification, dramatically reduces required time and input cell numbers. In comparison with current ChIP-seq variants [3, 5–12], HT-ChIPmentation is technically simple, extremely rapid and widely applicable, being compatible with both very low cell number requirements and high-throughput applications.

## Results

The adapters introduced by Tn5 are covalently linked only to one strand of the tagmented DNA. The complete adapters, compatible with PCR amplification, are created through a subsequent extension reaction. With this in mind, we reasoned that performing adapter extension of tagmented bead-bound chromatin and high-temperature reverse crosslinking [6], would allow us to bypass the DNA purification step.

**Fig. 1:**
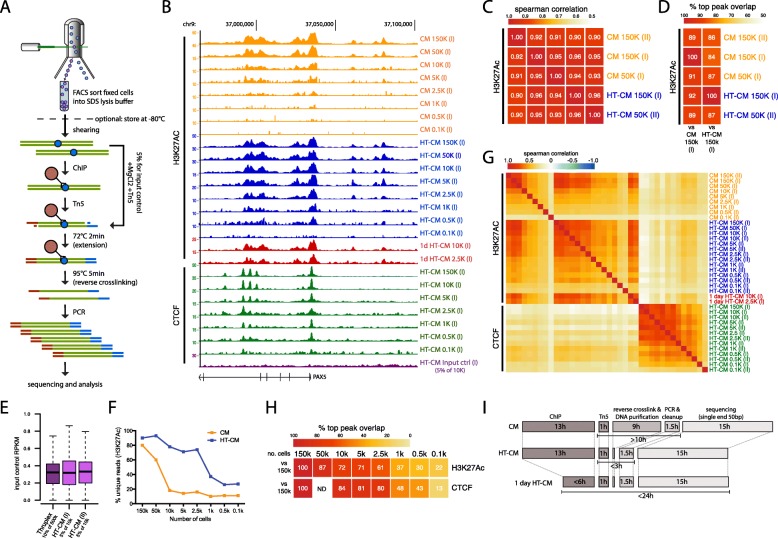
High-throughput ChIPmentation (HT-CM) through direct amplification of tagmented chromatin, allows for rapid and technically simple analysis of histone modifications and transcription factor binding in low numbers of FACS sorted cells. **a** Schematic overview of the HT-CM workflow (for a direct comparison between the HT-CM and original ChIPmentation (CM) methods, see Additional file 1: Figure S1). In brief, FACS sorted cells are sonicated, subjected to ChIP and tagmented. Library amplification is subsequently done without prior DNA purification. Input controls are prepared through direct tagmentation of sonicated chromatin. **b** Genome-browser profiles from CM, HT-CM and input control samples generated using indicated cell-numbers and antibodies. **c** Correlation between H3K27Ac signals (in a merged catalog containing all peaks identified in displayed samples) generated using indicated methods and cell numbers. **d** Overlap (%) between top peaks (peaks with the 50% highest peak quality scores) identified in high cell-number (150 and 50 k) H3K27Ac HT-CM and CM samples. **e** RPKM of 1 kb bins covering the whole genome in input control samples generated using indicated method and cell-equivalents of chromatin. **f** Percentage of unique reads in H3K27Ac HT-CM and CM samples generated in parallel. **g** Correlation between H3K27Ac/CTCF signals in samples generated using indicated methods and cell-numbers. **h** Overlap (%) between top peaks identified in H3K27Ac and CTCF HT-CM samples generated using indicated cell-numbers. ND, not done. **i** Time required to perform ChIP, library preparation and sequencing for the CM, HT-CM and 1-day HT-CM workflows. Hours (h) needed to perform each step are indicated

To validate this approach and benchmark it against standard ChIPmentation (Fig. 1a and Additional file 1: Figure S1), we FACS sorted defined numbers of formaldehyde fixed cells and performed ChIP with subsequent library preparation on cell numbers ranging from 0.1 to 150 k cells. HT-ChIPmentation indeed produced excellent sequencing profiles (Fig. 1b), and a consistent library size over > 100-fold difference in input cell numbers (Additional file 1: Figure S2A).

Looking specifically at H3K27Ac (a histone modification demarcating active promoters and enhancers [13]) HT-ChIPmentation and ChIPmentation samples generated in parallel from high cell-numbers (50–150 k cells), both methods generated high-quality data that is comparable in regard to: concordance of library profiles (Fig. 1b); mappability of sequencing reads (Additional file 1: Table S1); correlation between samples (Fig. 1c); number, quality scores and signal range of identified peaks (Additional file 1: Figure S2B–D); and peak overlap (Fig. 1d).

To perform accurate peak calling, input controls were generated by direct tagmentation of 500 cell equivalents of sonicated chromatin (5% of 10 k sonicated cells), subsequently processed in parallel with corresponding 10 k HT-ChIPmentation samples (Fig. 1a). The HT-ChIPmentation compatible input controls produced similar results as input controls prepared using traditional library preparation methodology, in terms of library profiles and even genomic coverage (Fig. 1b and e).

We next compared H3K27Ac HT-ChIPmentation and ChIPmentation samples from progressively lower input cell-numbers. As expected, eliminating losses associated with DNA purification allowed HT-ChIPmentation samples to maintain much higher library complexity (> 75% unique reads down to 2.5 k cells) than ChIPmentation samples generated from the same number of cells (Fig. 1f). This difference in library quality was directly reflected in HT-ChIPmentation samples generated from a few thousand cells maintaining: consistent high quality library profiles (Fig. 1b); mappability (Additional file 1: Table S1); number, quality scores and signal range of identified peaks (Additional file 1: Figure S2B–D); high correlation between samples (Fig. 1g); and high peak overlap (Fig. 1h). Similar results were obtained for H3K27Ac HT-ChIPmentation data generated in a single day (Fig. 1b, g and Additional file 1: Figure S2B–D). Based on the same metrics, CTCF (a chromatin organizing protein [14]) HT-ChIPmentation experiments further verified the robustness of the method with cell numbers in the range of a few thousands cells (Fig. 1b, g, h; Additional file 1: Figures S2B–D and S3A–B).

## Discussion

Here we present HT-ChIPmentation, an improved and simplified tagmentation based approach to produce ChIP-seq libraries. We demonstrate that the adapters introduced by Tn5 can be extended directly on the bead-bound chromatin. Through this, we can combine ChIPmentation [3] with high-temperature reverse crosslinking and direct library amplification without prior DNA purification [6]. Even compared to the already technically simple and fast ChIPmentation method, HT-ChIPmentation is easier to perform and greatly reduces the time needed to produce sequencing ready libraries (Fig. 1i). In fact, HT-ChIPmentation together with sequencing can be performed in a single day (Fig. 1b, g and i; Additional file 1: Figure S2B–D). This makes the protocol ideal for rapid data generation and compatible with the development of clinical diagnostic/prognostic applications relying on chromatin associated features to distinguish, for example, tumor subtypes [15, 16].

The removal of the DNA purification step, allows for fully taking advantage of that tagmentation of chromatin – as opposed to traditional adapter ligation [6, 8] – remains highly effective even with very limited input material ([3] and Additional file 1: Figure S2A). Together, the reduced losses of material and effective addition of adapters, allows HT-ChIPmentation to be performed on just a few thousand FACS sorted cells with maintained quality and library complexity. Hence, HT-ChIPmentation provides a robust and technically simple workflow for characterizing epigenetic changes and transcription factor binding in rare subsets of cells.

Input controls are commonly used to exclude biases in the input material and as a negative control for identification of peak regions. Here we show that input controls can be prepared in parallel with HT-ChIPmentation samples, through direct tagmentation and library amplification of sonicated chromatin. The protocol requires very limited material (500 cell equivalents of sonicated chromatin), making it both feasible and convenient to directly prepare adequate controls for peak finding, also from rare subsets of cells.

The simplicity of the HT-ChIPmentation protocol – allowing for performing all steps from cells to amplified sequencing ready library without DNA purification – makes it perfectly suited for epigenetic characterization at any scale. While HT-ChIPmentation is directly compatible with full automation, experiments presented here were simply performed in 96-well plates using a multi-channel pipette, demonstrating that HT-ChIPmentation makes it highly feasible to perform epigenome scale projects in a matter of days using standard laboratory equipment.

## Conclusion

Here we introduce HT-ChIPmentation, an improved tagmentation based ChIP-seq protocol that through the extension of the Tn5-inserted adapters on bead-bound chromatin, allows for direct library amplification without prior DNA purification. In comparison to current state-of-the-art ChIP-seq protocols [3, 5–12], HT-ChIPmentation is technically simple, extremely rapid and widely applicable, being compatible with very low cell number requirements, high-throughput applications and single day data generation. Taken together, HT-ChIPmentation provides a versatile and simplistic workflow attractive as the mainstay protocol for epigenome projects of any scale.

## Methods

### Cells

Cultured MEC1 cells were stained with LIVE/DEAD fixable Aqua stain (Invitrogen) to allow for excluding cells dead already prior to fixation (during subsequent FACS sorting) and fixed using 1% PFA (Pierce). Aliquots of 10 k cells were FACS sorted directly into 100 μl SDS lysis buffer (50 mM Tris/HCl, 0.5% SDS, and 10 mM EDTA) supplemented with 1X cOmplete EDTA-free protease inhibitor (Roche) and stored at − 80 °C until use. For aliquots of cells (50 and 150 k), where the sheath fluid volume is non-negligible, cells were sorted into PBS, spun down (2000 g 5 min) and resuspended in 100 μl SDS lysis buffer prior to freezing. Sorting was performed using a BD FACSAriaIIu cell sorter (BD Biosciences) with an 85 μm nozzle.

### Chromatin immunoprecipitation and tagmentation

For ChIP, polyclonal anti-H3K27Ac (Diagenode, cat# C15410196, lot# A1723-0041D) antibody or anti-CTCF (Diagenode, cat# C15410210, lot# A2359-00234P) antibody was added to Protein G-coupled Dynabeads (ThermoFisher) in PBS with 0.5% BSA and incubated with rotation for 4 h at 4 °C (0.5 h at RT for HT-ChIPmentation samples processed in a single day). For 50–150 k cells, 10 μl beads incubated with 3 μg H3K27Ac or 1.5 μg CTCF antibody were used per ChIP. For 0.1–10 k cells, 2 μl beads incubated with 0.6 μg H3K27Ac or 0.3 μg CTCF antibody were used per ChIP. Fixed cells (FACS sorted) frozen in SDS lysis buffer were thawed at room temperature. To perform ChIP on < 10 k cells, aliquots were diluted with SDS lysis buffer and 100 μl containing the appropriate number of cells were processed. Cells were sonicated for 12 cycles of 30 s on/30 s off on high power using a Bioruptor Plus (Diagenode). To neutralize the SDS, Triton X100 was added to a final concentration of 1% along with 2 μl 50x cOmplete protease inhibitor (final 1x). Samples were incubated at room temperature for 10 min and when applicable 5% aliquots were saved for preparation of input controls. Antibody-coated Dynabeads were washed with PBS with 0.5% FCS and mixed with cell lysate in PCR tubes. Tubes were incubated rotating overnight (or 4 h for HT-ChIPmentation samples processed in a single day) at 4 °C.

Immunoprecipitated chromatin was washed with 150 μl of low-salt buffer (50 mM Tris/HCl, 150 mM NaCl, 0.1% SDS, 0.1% NaDOC, 1% Triton X-100, and 1 mM EDTA), high-salt buffer (50 mM Tris/HCl, 500 mM NaCl, 0.1% SDS, 0.1% NaDoc, 1% Triton X-100, and 1 mM EDTA) and LiCl buffer (10 mM Tris/HCl, 250 mM LiCl, 0.5% IGEPAL CA-630, 0.5% NaDOC, and 1 mM EDTA), followed by two washes with TE buffer (10 mM Tris/HCl and 1 mM EDTA) and two washes with ice cold Tris/HCl pH 8. For tagmentation, bead bound chromatin was resuspended in 30 μl of tagmentation buffer, 1 μl of transposase (Nextera, Illumina) was added and samples were incubated at 37 °C for 10 min followed by two washes with low-salt buffer.

### High-throughput ChIPmentation library preparation

For High-throughput ChIPmentation (HT-CM) samples, bead bound tagmented chromatin was diluted in 20 μl of water. PCR master mix (Nextera, Illumina) and indexed amplification primers [17] (0.125uM final concentration) was added and libraries prepared using the following program: 72 °C 5 min (adapter extension); 95 °C 5 min (reverse cross-linking); followed by 11 cycles of 98 °C 10s, 63 °C 30s and 72 °C 3 min.

For preparation of HT-CM compatible input controls, 1 μl of 50 mM MgCl_2_ was added to 5 μl sonicated lysate (5% aliquot of 10 k samples) to neutralize the EDTA in the SDS lysis buffer. Thirty microliters of tagmentation buffer and 1 μl transposase (Nextera, Illumina) was added, and samples were incubated at 37 °C for 10 min. 22.5 μl of the transposition reaction were combined with 15 μl of PCR master mix and 2.5 μl of primer mix (Nextera, Illumina). Libraries were subsequently amplified as described for HT-ChIPmentation samples.

### ChIPmentation library preparation

For standard reverse crosslinking, chromatin complexes were diluted with 200 μl ChIP elution buffer (10 mM Tris/HCl, 0.5% SDS, 300 mM NaCl, and 5 mM EDTA) and 2 μl of 20 μg/ml proteinase K (Thermo Scientific). Samples were vortexed and incubated with shaking overnight at 65 °C. After reverse crosslinking, 1 μl 20 μg/ml RNase (Sigma) was added and incubated at 37 °C for 30 min. After another 2 h of incubation with 2 μl of proteinase K (20 mg/ml) at 55 °C, samples were placed in a magnet to trap magnetic beads and supernatants were collected. DNA purification was carried out using Qiagen MinElute PCR Purification Kit. Fifteen microliters of PCR master mix and 5 μl of primer mix (Nextera, Illumina) was added to 20 μl of eluted DNA, and libraries were amplified as described for HT-ChIPmentation libraries.

### Preparation of conventional input control

Sonicated material from 50 k cells was reverse crosslinked as described for ChIPmentation. Two nanograms of DNA was used for library preparation using the ThruPLEX DNA-seq kit (Rubicon Genomics) with 11 cycles of PCR amplification.

### Post-PCR library cleanup and sequencing

After PCR amplification, library cleanup was done using Agencourt AmPureXP beads (Beckman Coulter) at a ratio of 1:1. DNA concentrations in purified samples were measured using the Qubit dsDNA HS Kit (Invitrogen). Libraries were pooled and single-end sequenced (50 cycles) using the Nextseq500 platform (Illumina).

### Basic processing of ChIP-seq and input control sequencing data

Quality of the sequenced samples was assessed using FastQC v0.11.5 [18]. Samples were mapped to the human reference genome (hg19) using Bowtie2 v2.2.3 [19] with default settings. Further basic processing was performed using HOMER v4.8.3 [20]. Specifically, mapped reads were converted into tagdirectories by the makeTagDirectory command using settings for the human genome (-genome hg19) and removing duplicate reads by allowing only one tag to start per base pair (-tbp 1).

### Genome browser visualizations

Bedgraphs were created for each sample using HOMER’s makeUCSCfile. Tracks were uploaded and visualized using the UCSC genome browser [21].

### Peak finding and plotting peak metrics

Peak finding was performed using the findPeaks command in HOMER. Peaks were called using default settings for histone modifications (-style histone) and transcription factors (-style factor) for H3K27Ac and CTCF respectively with input (-i) as a control. Visualization was done in R v3.1.0 [22], using the built in barplot and boxplot R-functions to plot peak numbers and peak quality scores, respectively.

### Making and annotating peak catalogs

Peak catalogs were created by merging all peak files of samples analyzed using HOMER’s mergePeaks command. Setting used (-size given) ensured that peaks with literal overlap were merged to one peak while peaks unique to one sample were directly added to the peak catalog. Subsequently, peak catalogs were annotated with unnormalized (-raw) read counts within peaks in the catalog for each individual sample using HOMER’s annotatePeaks.pl script.

### Plotting peak read distributions and correlation between samples

Raw counts were log normalized in R as follows: log(df[,countsCols]+1,2). Log2 counts were subsequently plotted using the build in boxplot R-function. These same Log2 counts were used to calculate sample correlations, using the build-in cor R-function with spearman correlation. Correlation matrices were visualized with the pheatmap function from the pheatmap R-package using color scales generated with the build-in colorRampPalette R-function.

### Plotting reads within 1 kb bins for input control samples

A file containing 1 kb bins covering the whole genome was created using the makewindows command from bedtools v2.26.0 [23] using a window size of 1 kb (-w 1000). Chromosome sizes were retrieved as follows: mysql --user=genome --host=genome-mysql.cse.ucsc.edu -A -e "select chrom, size from hg19.chromInfo" > hg19.genome. Raw reads in each 1 kb bin for each input control were counted using HOMER’s annotatePeaks.pl script, as described above. Raw read distributions were converted to RPKM in R based on the standard RPKM formula. Resulting RPKM distributions were plotted with the build-in boxplot R-function.

### Determining top peak overlap

Peaks identified in individual samples were overlapped with in-house code using the IRanges [24] R-package. Top peaks overlap was considered to be the percentage of high quality peaks (50% of peaks with highest quality scores) in the reference sample that overlap (≥1 bp) with a peak in the second sample. For purposes of determining peak overlap, CTCF peaks were extended with 50 bp up and downstream, considering findPeaks with -style factor only calls a small region around the peak maximum. Peak overlaps were visualized using the pheatmap function from the pheatmap R-package using color scales generated with the build-in colorRampPalette R-function.

### Comparing library complexity

To compare duplication rates between HT-ChIPmentation and ChIPmentation samples, fastq files were randomly down-sampled to the total number of reads in the smallest file for each cell number. Down sampling was performed using the fastq-sample script from fastq-tools v0.8 [25]. Fraction of unique reads was subsequently determined for each file using FastQC v0.11.5.

### Motif enrichment analysis

Enrichments of known transcription factor binding motifs in peaks were identified using HOMER’s findMotifsGenome.pl script with default settings.

## Additional file

Additional file 1: **Figure S1.** Schematic comparison of the ChIPmentation and high-throughput ChIPmentation protocols. **Figure S2.** High-throughput ChIPmentation (HT-CM) samples maintain library quality over progressively lower input cell numbers. **Figure S3.** High-throughput ChIPmentation (HT-CM) maintains high library complexity in CTCF samples. **Table S1.** Sample details. (PDF 323 kb)

## Abbreviations

ChIP-seq: Chromatin immunoprecipitation with high-throughput sequencing
CM: ChIPmentation
HT-ChIPmentation: High-throughput ChIPmentation
HT-CM: High-throughput ChIPmentation
