# Supplementary material for: High-throughput ChIPmentation: freely scalable, single day ChIPseq data generation from very low cell-numbers
Source: BMC Genomics. 2019 Jan 18;20:59. doi: 10.1186/s12864-018-5299-0 (PMC6339279; doi:10.1186/s12864-018-5299-0)
Supplement: Supplementary file 1 — Figure S1. Schematic comparison of the ChIPmentation and high-throughput ChIPmentation protocols. Figure S2. High-throughput ChIPmentation (HT-CM) samples maintain library quality over progressively lower input cell numbers. Figure S3. High-throughput ChIPmentation (HT-CM) maintains high library complexity in CTCF samples. Table S1. Sample details. (PDF 323 kb) [file 12864_2018_5299_MOESM1_ESM.pdf]

Additional File 1 contents:

Supplemental Figure 1. Schematic comparison of the ChIPmentation and high-throughput ChIPmentation protocols.

Supplemental Figure 2. High-throughput ChIPmentation (HT-CM) samples maintain library quality over progressively lower input cell numbers.

Supplemental Figure 3. High-throughput ChIPmentation (HT-CM) maintains high library complexity in CTCF samples

Supplemental Table 1. Sample details

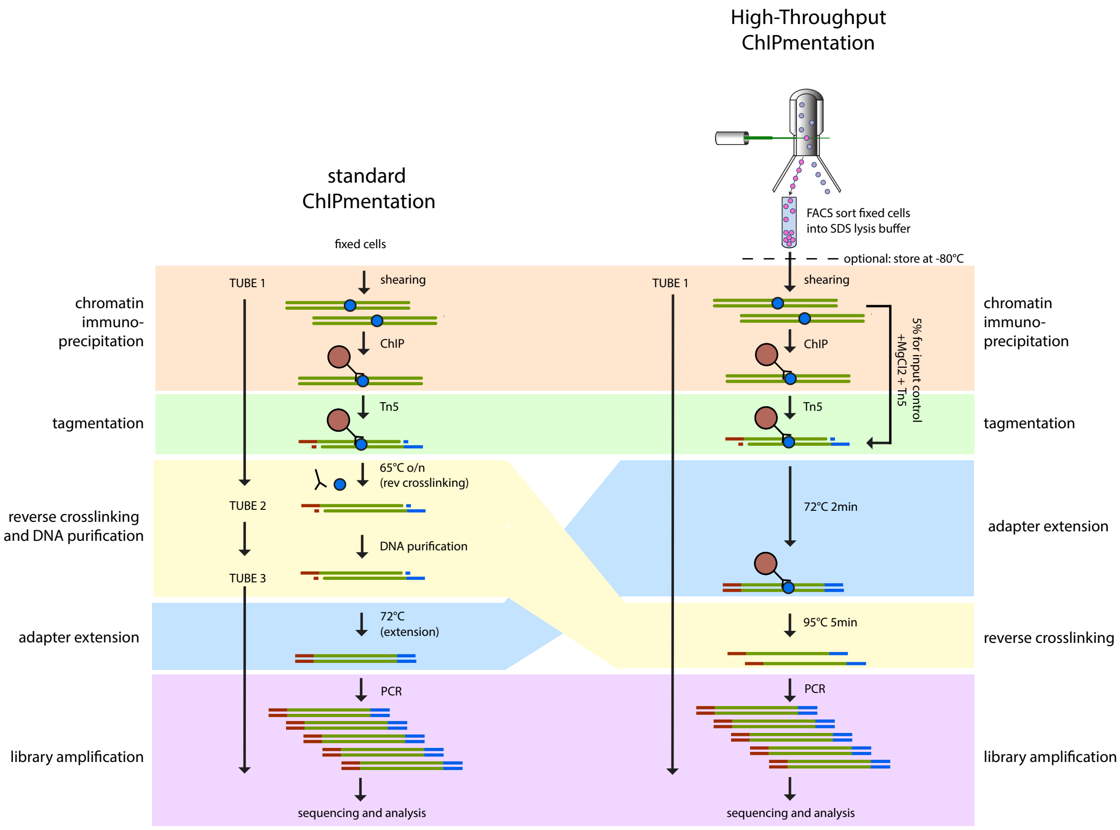

Supplemental Figure 1. Schematic comparison of the ChIPmentation and High-Throughput (HT) ChIPmentation protocols. Immunoprecipitation and tagmentation of chromatin is done in the same way for both protocols. Standard ChIPmentation (left) utilize traditional reverse crosslinking followed by DNA purification and PCR amplification while HT-ChIPmentation (right) directly performs adapter extension and reverse crosslinking as part of the library amplification.

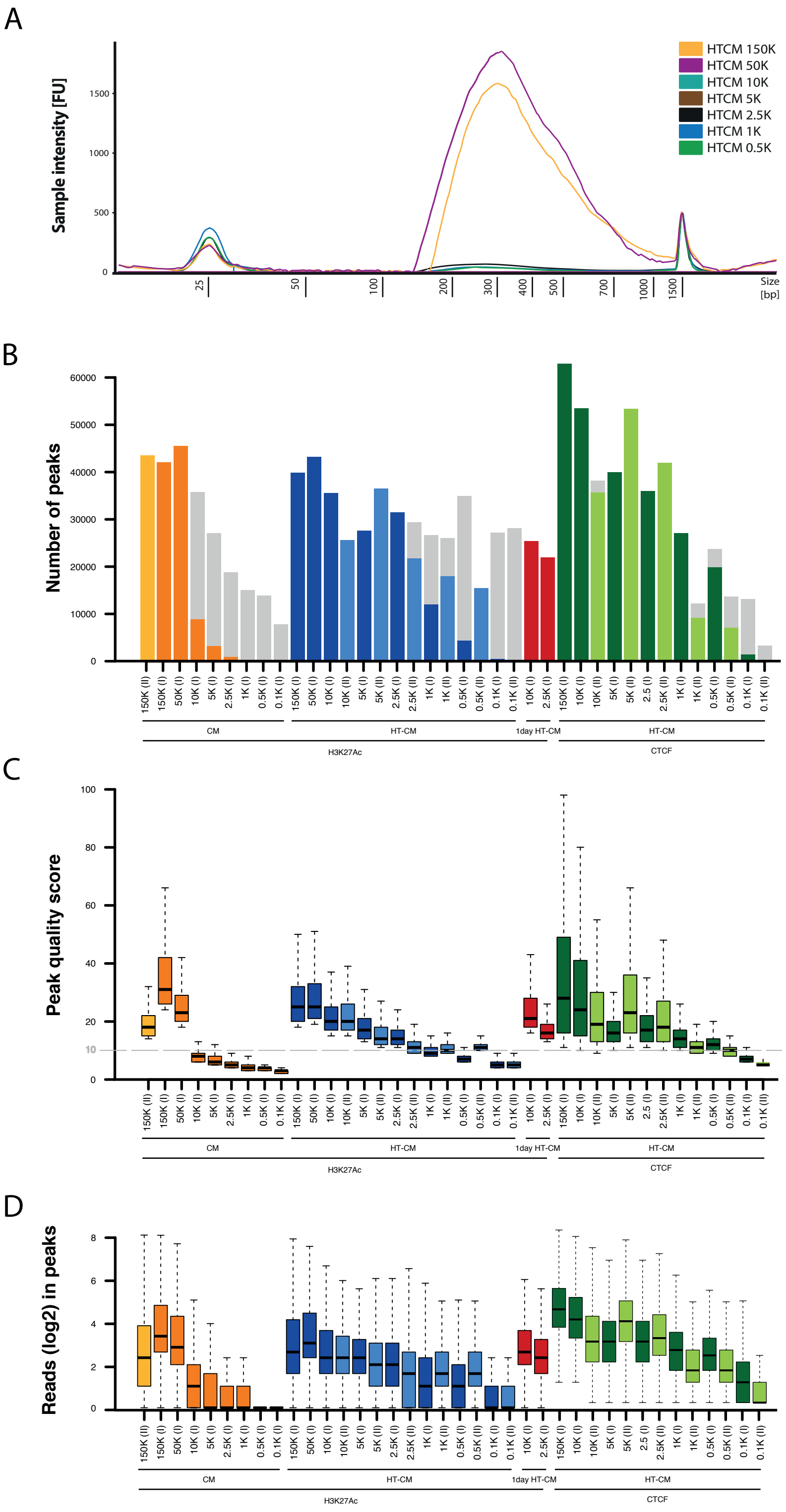

Supplemental Figure 2. High-throughput ChIPmentation (HT-CM) samples maintain library quality over progressively lower input cell numbers. a) Tape station traces showing relative amounts and library size of H3K27Ac HT-ChIPmentation samples from indicated cell numbers. b) Distribution of reads (log2) within peaks of indicated samples. Reads were quantified in a merged peak catalog containing all peaks identified in H3K27Ac or CTCF samples respectively. c) Peak quality score distribution in CM and HT-CM samples generated using indicated cell numbers and antibodies. Grey line indicates the quality score cut-off (10) used in d). d) Number of identified peaks in CM and HT-CM samples generated using indicated cell numbers and antibodies. Bars indicate the number of identified high quality peaks. Grey indicates identified low quality peaks (quality score <10).

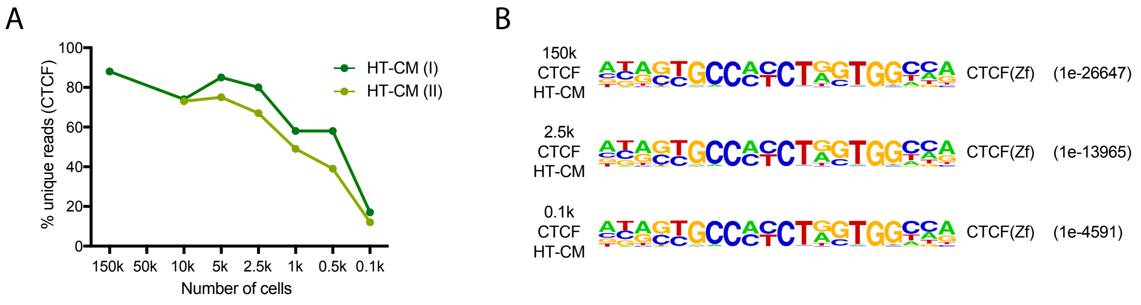

Supplemental Figure 3. High-throughput ChIPmentation (HT-CM) maintains high library complexity in CTCF samples. a) Percentage of unique reads in CTCF HT-ChIPmentation samples. HT-CM (I) and (II) indicate data from separate experiments. b) The most significantly enriched motif (identified by HOMER) found in HT-CM CTCF peaks from indicated cell numbers are shown. Motif enrichment p-values are indicated in parenthesis.

Antibody	Sample	DNA yield (ng)	# total reads sequenced	# total mapped reads	% total mapped reads	# unique positions in Homer tag directory	estimated % unique reads (determined by FastQC)
H3K27Ac	CM 150K (II)	0.5	24092055	23806655	99	19289577	88
	CM 150K (I)	25.7	87516317	85819989	98	45196491	80
	CM 50K (I)	5.5	121849142	119584399	98	28718618	60
	CM 10K (I)	0.7	91589798	89477891	98	3552803	18
	CM 5K (I)	0.5	77861279	75607160	97	2088343	14
	CM 2.5K (I)	2.2	13895814	13540068	97	1223246	16
	CM 1K (I)	0.5	18917603	18339194	97	638941	10
	CM 0.5K (I)	0.7	16851154	16306991	97	642112	11
	CM 0.1K (I)	0.5	5520137	5293533	96	136176	11
	HT-CM 150K (I)	190.0	35198189	34244529	97	28989575	90
	HT-CM 50K (I)	305.0	38594146	37734305	98	32960974	93
	HT-CM 10K (I)	22.3	30665999	29496188	96	21352892	78
	HT-CM 10K (II)	8.8	65560037	60958211	93	22027207	61
	HT-CM 5K (I)	14.6	28973434	27599052	95	17849828	71
	HT-CM 5K (II)	5.2	64437331	62328094	97	13796228	46
	HT-CM 2.5K (I)	6.8	26532665	25363846	96	13836102	74
	HT-CM 2.5K (II)	2.9	58693804	56060570	96	8120134	51
	HT-CM 1K (I)	1.6	27783412	26196310	94	6183269	37
	HT-CM 1K (II)	6.0	37883698	36403614	96	9029360	48
	HT-CM 0.5K (I)	1.3	18478771	16439432	89	3299219	26
	HT-CM 0.5K (II)	3.3	83006733	77717188	94	9858925	51
	HT-CM 0.1K (I)	0.5	13961187	12663224	91	1205248	27
	HT-CM 0.1K (II)	0.9	22853683	20256301	89	1235808	27
	HT-CM OneDay 10K	4.9	47387610	45551060	96	25600123	74
	HT-CM OneDay 2.5K	3.5	50183020	47834659	95	16961174	73
CTCF	HT-CM 150K (I)	222.0	50919867	49265841	97	39126041	88
	HT-CM 10K (I)	20.2	48258003	46844722	97	30417442	74
	HT-CM 10K (II)	17.3	35213122	33733362	96	21803169	73
	HT-CM 5K (I)	71.8	34410858	33423150	97	25073425	85
	HT-CM 5K (II)	25.5	51545017	49393971	96	33164149	75
	HT-CM 2.5K (I)	30.0	44297358	41678227	94	29186558	80
	HT-CM 2.5K (II)	12.9	50157190	47236609	94	27771813	67
	HT-CM 1K (I)	8.7	48718267	46621748	96	22112712	58
	HT-CM 1K (II)	6.2	42425230	39423550	93	16195688	49
	HT-CM 0.5K (I)	11.1	47395951	44656741	94	21128432	58
	HT-CM 0.5K (II)	4.2	49282361	45309121	92	14225428	39
	HT-CM 0.1K (I)	1.9	79280567	70597496	89	5776648	17
	HT-CM 0.1K (II)	0.6	48760160	34677569	71	2142994	12
Input	Thruplex 10% of 500K	78.9	38487512	37261151	97	30921685	93
	HT-CM 5% of 10K (I)	14.5	100318331	88806740	89	66047527	77
	HT-CM 5% of 10K (II)	5.8	66984603	57863640	86	45201303	86

Supplemental Table 1. Sample details. Library yield, total reads, mappability and unique reads are given for ChIPmentation (CM) and High-throughput ChIPmentation (HT-CM) samples. (I) and (II) indicate experimental replicas.
